# Preparation and Compression Resistance of Lightweight Concrete Filled with Lightweight Calcium Carbonate Reinforced Expanded Polystyrene Foam

**DOI:** 10.3390/polym15244642

**Published:** 2023-12-08

**Authors:** Erke Wang, Lixue Xiang, Bo Tang, Xuming Dai, Zheng Cao, Tao Jiang, Ying Wang, Xiaowen Chen, Wenge Li, Yuantao Zhao, Ke Yang, Xinfeng Wu

**Affiliations:** 1 Shanghai Key Laboratory of Engineering Materials Application and Evaluation, China Shanghai Thermophysical Properties Big Data Professional Technical Service Platform, Shanghai Engineering Research Center of Advanced Thermal Functional Materials, School of Energy and Materials, Shanghai Polytechnic University, Shanghai 201209, China; wx@vulcan-hz.com; 2 Hangzhou Vulcan New Materials Technology Co., Ltd., Hangzhou 311255, China; xianglixue@vulcan-hz.com (L.X.); luoyahangzhou@163.com (B.T.); dxm01@vulcan-hz.com (X.D.); 3 Merchant Marine College, Shanghai Maritime University, Shanghai 201306, China; caozheng96@163.com (Z.C.); jiangtao9585@163.com (T.J.); 201940110009@stu.shmtu.edu.cn (Y.W.); wgli@shmtu.edu.cn (W.L.); zhaoyt@shmtu.edu.cn (Y.Z.); 4 School of Materials & Enviromental Engineering, Shenzhen Polytechnic University, Shenzhen 518055, China; 5 College of Materials Science and Engineering, Central South University, Changsha 410000, China; keyang@csu.edu.cn

**Keywords:** lightweight calcium carbonate, lightweight concrete, compression strength, low density

## Abstract

Lightweight concrete is widely used in the construction industry due to its low density and high strength. In this paper, lightweight concrete was prepared by a simple two-step method. Firstly, the light calcium carbonate reinforced epoxy macrospheres (LCR-EMS) material was obtained by adhering calcium lighter carbonate powder to the expanded polystyrene foam spheres (EPS) material using the “balling method”. In the second step, the LCR-EMS was mixed with water, cement, and the hollow glass microspheres (HGMS) material using the “molding method” to obtain lightweight concrete. The combination of macroscopic photographs and microscopic morphology shows that the LCR-EMS material itself is uniformly encapsulated and well bonded to the matrix. Test results show that the density of the lightweight concrete decreases with an increase in the volume fraction of stacked LCR-EMS, the diameter, and the proportion of HGMS in the matrix, but it decreases with a decrease in the number of layers of LCR-EMS. The compressive strength of lightweight concrete exhibits a completely opposite trend. When three layers of LCR-EMS were used as filler material, the density and compressive strength of the concrete were 1.246 g/cm^3^ and 8.19 MPa, respectively. The density and maximum compressive strength of lightweight concrete were 1.146 g/cm^3^ and 6.37 Mpa, respectively, when filled with 8–9 mm-2L-90 svol% of LCR-EMS and 40 wt% of HGMS in the matrix. Compared with lightweight concrete filled with 90% EPS, the density increased by 20% while the compressive strength increased by 300%.

## 1. Introduction

The building materials industry is an important basic industry of the national economy and provides support for improving living conditions, managing the ecological environment, and developing a circular economy [1,2]. The building materials industry is a key area of industrial energy consumption, carbon emissions, and an important sector in achieving the “double carbon” goal. Lightweight concrete is a special type of concrete that has a relatively low density, usually half or less than that of ordinary concrete. Lightweight concrete is used in a wide range of applications, including building walls, insulation, roofing, and foundation filling. Due to its low density, high seismic performance, and low energy consumption, lightweight concrete can replace original concrete materials, thus promoting the low-carbon development of the construction industry [3,4,5].

At the present stage, the preparation of lightweight concrete mainly includes the foaming method and filling method. The foaming method mainly involves the introduction of a foaming agent to induce foaming [6,7]. The core of the foaming method is the use of blowing agents, which can be chemical blowing agents or physical blowing agents. Chemical blowing agents create air bubbles in the concrete, while physical blowing agents introduce air bubbles through mechanical action [8,9]. Foamed lightweight concrete has advantages such as lightness, insulation, and environmental friendliness, but it also has disadvantages, such as a low compressive strength and uneven density [10,11]. Filled lightweight concrete is made by adding lightweight fillers directly or indirectly modified into the concrete and mold [12,13]. At present, fillers for lightweight concrete mainly include expanded perlite [14,15], foam particles (such as polystyrene foam balls [16,17], ceramic balls [18,19], and so on), and expanded clay [20,21]. Stefania Grzeszczyk et al. [22] added expanded perlite to concrete to obtain lightweight concrete. Test results showed that the density of lightweight concrete was 1900 kg/m^3^, and the compressive strength was >70 MPa when filled with 30 vol% expanded perlites. Also, the addition of expanded perlite can reduce the water absorption of lightweight concrete. Jiang et al. [23] used aerogel to reinforce polystyrene foam balls and filled them into lightweight concrete. The test results showed that when the matrix was filled with 2 wt% of 12 mm GF and 90% aerogel-reinforced polystyrene foam balls, the lightweight concrete met the requirements for high strength (11.46 MPa) and low density (0.897 g/cm^3^). Stefania Grzeszczyk et al. [24] investigated the effect of adding an expanded clay aggregate to concrete on the properties of composites. The test results showed that the compressive strength of lightweight concrete decreased from 75.2 MPa to 64.5 MPa, while the flexural strength increased from 12.4 MPa to 12.9 MPa when the addition content varied from 30 vol% to 60 vol%. The water absorption of lightweight concrete can be reduced to 3%. The addition of reinforcing foam balls to concrete makes it possible to construct the internal cellular structure of the concrete, so that lower-density concrete materials can be obtained. Materials for foam ball reinforcement can include cement, aerogel, fiber powder, calcium carbonate powder, and so on.

After powder treatment, calcium carbonate particles become smaller and, in the role of cement and water, will effectively increase the amount of slurry and the specific surface area of the slurry [25,26]. In the process of cement hydration, calcium carbonate powder particles can be used as nucleation sites, increasing the hydration product C-S-H gel precipitation on limestone powder particles of the index; at the same time, it can accelerate the hydration speed of C3S [27]. The hydration reaction between calcium carbonate and C3A forms a calcium carbon-aluminate, which can promote the hardening of cementitious materials. The mixing of calcium carbonate powder improves the compatibility of concrete, reducing voids or pores in the concrete, thus making the coarse aggregate play a greater role in the stress process [28,29]. Calcium carbonate powder can be divided into heavy calcium carbonate [30], light calcium carbonate, nano calcium carbonate [31], and so on, according to density. Light calcium carbonate involves removing some or all calcium carbonate crystals, forming light particles with a porous structure. Unlike heavy calcium carbonate powder, light calcium carbonate powder has a more regular particle shape and a narrower particle size distribution.

In this paper, lightweight concrete composites filled with lightweight calcium carbonate reinforced EPS (LCR-EMS) were prepared and obtained by the combination of tumbling and molding methods. The test results show that the density of the lightweight concrete increases with the increase in the stacking volume fraction, diameter, and percentage of HGMS in the matrix, but decreases with the decrease in the number of layers of LCR-EMS. The trend of the compressive strength of the lightweight concrete was completely opposite. The density and strength of the overall material can be controlled by adjusting the ratio, size, and diameter of the LCR-EMS in the lightweight concrete.

## 2. Materials and Methods

### 2.1. Materials

Epoxy resin (Araldite^®^LY 1564) and amine curing agent (Aradur^®^3486) were purchased from Huntsman Chemical Co., Ltd., The Woodlands, TX, USA. Regarding these materials, the bisphenol A-type epoxy resin was a colorless transparent liquid with a viscosity of 1200–1400 mPa-s at 25 °C, a density of 1.1–1.2 g/cm^3^, and an epoxy index (ISO 3001 [32]) of 5.80–6.05 eq/kg. The amine curing agent was a light yellow liquid with a viscosity range of 10–20 mPa-s at 25 °C, a density of 0.94–0.95 g/cm^3^, and an amine value (ISO 9702 [33]) of 8.55–9.30 eq/kg. Light calcium carbonate was purchased from Shandong Rongwei Chemical Co., Ltd., Jinan, China. The particle size of light calcium carbonate powder was 40–50 μm. The 42.5 Portland cement was purchased from Shenzhen Zhongning Technology Co., Ltd., Shenzhen, China. Expanded polystyrene foams (EPS) are produced in Hangzhou Hangchao Packaging Materials Co., Ltd., Hangzhou, China, with a diameter of 8–11 mm. Hollow glass microspheres (HGMS) material of type K1 was purchased from the 3M Company in St. Paul, MN, USA. The compressive strength of K1 was 1.72 MPa, the true density was 0.125 g/cm^3^, and the particle size was 30~120 μm.

### 2.2. Preparation Process of LCR-EMS and Characterization

**Figure 1:**
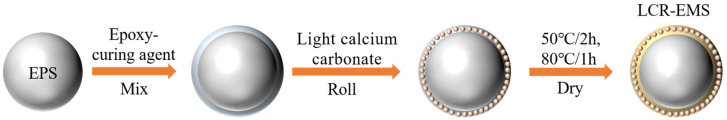
Schematic diagram of the preparation process of LCR-EMS.

Figure 1 is a schematic diagram of the preparation process of LCR-EMS. From the figure, it can be seen that the preparation process of LCR-EMS is mainly divided into three steps. In the first step, EPS is added to the epoxy hardener system so that its surface is completely covered by the resin. In this experiment, the ratio of epoxy resin to hardener was 3:1. For bisphenol A-type epoxy resin, amine hardener, and EPS, the masses used were 39 g, 13 g, and 5 g, respectively. In the second step, the lightweight concrete powder was uniformly wrapped around the surface of EPS by the “rolling ball method”. The lightweight calcium carbonate powder must be in excess to ensure that each EPS is completely encapsulated by the powder. In the third step, the preformed LCR-EMS was placed in an oven for curing with stepwise heating. The heating temperatures were 50 °C for 2 h and 80 °C for 1 h. Afterwards, the LCR-EMS with completed high temperature curing was subjected to room-temperature curing for more than 24 h.

**Figure 2:**
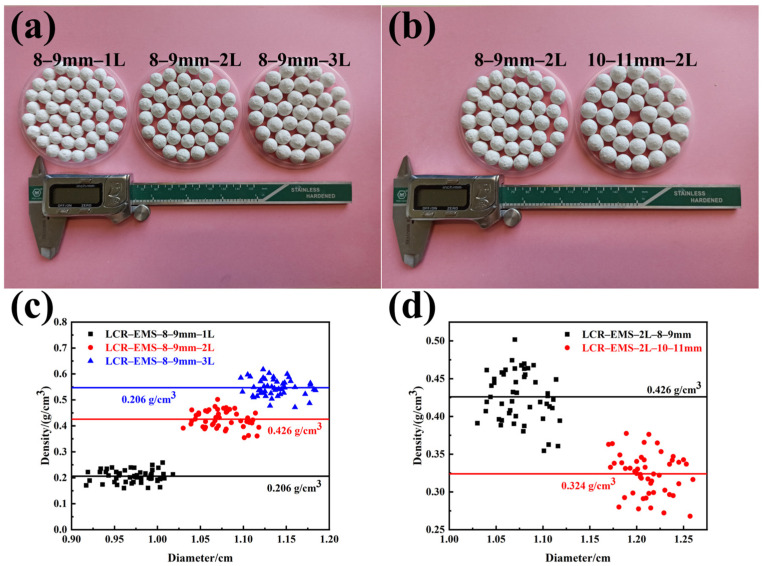
Macroscopic photographs and the density characterization of LCR-EMS. (**a**) Photographs of LCR-EMS with a different number of layers. (**b**) Photographs of LCR-EMS with different diameters in the same number of layers. (**c**) Diameter and density distribution graph of LCR-EMS in different layers. (**d**) Diameter and density distribution graph of LCR-EMS with different diameters at the same number of layers.

Figure 2 shows the macroscopic photographs and density characterization of LCR-EMS. From Figure 2a,b, it can be seen that the surface of LCR-EMS is more uniformly wrapped and the spheres are regular. Figure 2c,d show the relationship between the diameter and density of LCR-EMS. In order to avoid random data and reduce the error as much as possible, 50 spheres of LCR-EMS were randomly selected from each group for mass and diameter measurements. From Figure 2c, it can be seen that when the initial diameter of EPS beads is in the range of 8-9 mm, the densities of LCR-EMS with different layers are mainly distributed in three regions: 0.160–0.259 g/cm^3^ (LCR-EMS-1L), 0.354–0.502 g/cm^3^ (LCR-EMS-2L), 0.472–0.618 g/cm^3^ (LCR-EMS-3L). This shows that both EPS diameter and number of wrapped layers play a positive role in LCR-EMS. The thicker LCR-EMS helps to increase its own compressive strength but also contribute to increase the density of the lightweight concrete. It can also be seen from the figure that the average densities of LCR-EMS with a different number of layers are 0.206 g/cm^3^ (1L), 0.426 g/cm^3^ (2L), and 0.547 g/cm^3^ (3L), respectively.

### 2.3. Preparation Process of Lightweight Concrete

**Figure 3:**
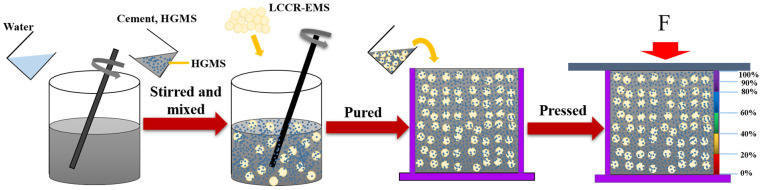
Schematic diagram of lightweight concrete preparation process.

Figure 3 shows a schematic diagram of the preparation process of lightweight concrete. In this paper, the preparation process is divided into three steps. First, the matrix filler is configured. LCR-EMS, cement, water, and HGMS were stirred and mixed, where the mass ratio of water to the sum of HGMS and cement was 1:3. The prepared initial samples were then poured into molds (70.7 mm × 70.7 mm × 70.7 mm). In order to reduce the generation of bubbles, defects, etc., during the filling process, pre-compression can be used to prepare the lightweight concrete. Finally, after 48 hours of compaction, the specimens were demolded and cured. The temperature of the curing box was controlled at 25 °C, the air humidity was 95%, and the curing time was 28 days. The main purpose of this experiment was to investigate the effects of various factors on the compressive properties of lightweight concrete. The influencing factors were mainly the stacking volume fraction, the diameter and number of layers of LCR-EMS, and the grading of HGMS and cement. The specific mix designs for the experiment are shown in Table 1. The filled HGMS is the mass percentage of matrix cement.

### 2.4. Characterization of Composites

A digital analytical balance (Guangzhou BGD Experimental Instrument Supply Company, Guangzhou, China) and digital caliper were used to measure the mass and diameter of the LCR-EMS, respectively, which were used to calculate to obtain the density of the LCR-EMS. The compressive strength of lightweight concrete was measured and analyzed using a universal testing machine (CMT5350, Shenzhen Sansi Zongheng Technology Co., Ltd., Shenzhen, China). This experiment is based on GB/T16491-2008 as the testing standard. Among them, the maximum test force of the universal testing machine is 300 KN and the maximum test range is 1100 mm. A scanning electron microscope (SEM) (JEM-4701, JEOL, Tokyo, Japan) was used to observe the bonding problem of the interface in lightweight concrete. The observed specimens were brittle cross sections.

## 3. Results and Discussion

### 3.1. Characterization of Density and Compressive Strength of Lightweight Concrete Filled with Different Stacking Volume Fractions

**Figure 4:**
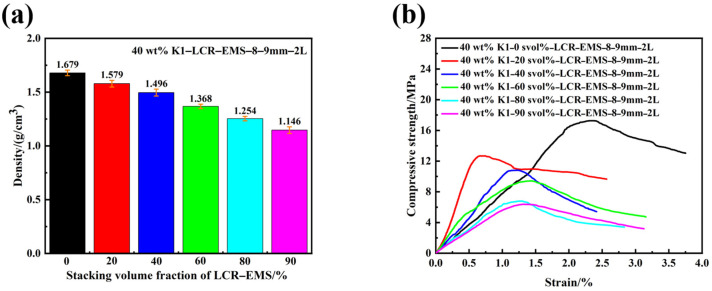
Density (**a**) and compressive strength (**b**) curves of lightweight concrete filled with different stacking volumes of LCR-EMS.

In order to investigate the effect of LCR-EMS with different stacking ratios on the compressive strength and density of lightweight concrete, we set five parameters with different stacking volume fractions of 20%, 40%, 60%, 80%, and 90%. Figure 4a shows the density of lightweight concrete filled with different stacking volume fractions of LCR-EMS. The density of lightweight concrete decreases with an increasing LCR-EMS stacking volume fraction, where the density of unfilled LCR-EMS lightweight concrete is 1.679 g/cm^3^ and the density of filled 90 svol% LCR-EMS lightweight concrete is 1.146 g/cm^3^. Figure 4b shows the compressive strength curves of lightweight concrete filled with different stacking volume fractions of LCR-EMS. The trend of compressive strength of lightweight concrete filled with different stacking volume fractions of LCR-EMS is the same as that of density. The higher the fraction volume of LCR-EMS, the lower the compressive strength of the concrete. In this case, the compressive strength of concrete filled with 90 svol% LCR-EMS is 6.37 MPa. The compressive strength decreases significantly compared to the concrete without LCR-EMS. The compressive strength of lightweight concrete decreased with the increase in the LCR-EMS stacking volume. When the LCR-EMS stacking volume is 20%, the decreasing trend of compressive strength of lightweight concrete is obvious, which indicates that the appropriate increase in the filling volume of LCR-EMS with a relatively small LCR-EMS stacking volume does not cause the destruction of the compressive strength of lightweight concrete. The maximum compressive strength of lightweight concrete was 6.79 MPa and 6.37 MPa when 80 svol% and 90 svol% LCR-EMS were added, respectively. There is a significant decrease in the maximum compressive strength of lightweight concrete (12.68 MPa) as compared to the maximum compressive strength of lightweight concrete with 20 svol% LCR-EMS. As the stacking volume fraction of LCR-EMS increases, the probability of their contacting each other in the matrix increases, and when the material is subjected to compressive loading, the stress concentration phenomenon occurs at the point of contact. The LCR-EMS forms a pore structure inside the material, which can effectively reduce the density of the composite material. However, LCR-EMS is also a type of defect and is more susceptible to stress damage compared to the matrix. In addition, an increase in LCR-EMS void volume not only tends to create bubbles during concrete preparation but also increases the likelihood of interfacial defects in the material.

### 3.2. Characterization of Density and Compressive Strength of Lightweight Concrete Filled with LCR-EMS of Different Diameters

**Figure 5:**
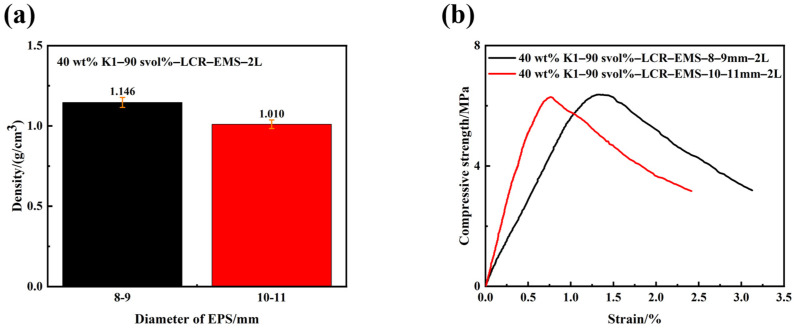
Density (**a**) and compressive strength (**b**) curves of lightweight concrete filled with different diameters of LCR-EMS.

Figure 5 shows the density and compressive strength curves of lightweight concrete filled with different diameters of LCR-EMS. From the figure, it can be obtained that the densities of the lightweight concrete are 1.146 g/cm^3^ (8–9 mm) and 1.010 g/cm^3^ (10–11 mm), and the corresponding maximum compressive strengths are 6.37 MPa and 6.29 MPa. The compressive strength of lightweight concrete decreases with an increasing inner diameter. This is mainly due to the fact that the smaller the diameter of the LCR-EMS, the faster the force transmission rate at its surface, that is, the faster the force diffusion rate. The faster the force diffusion rate, the faster the rate of stress concentration inside the concrete; therefore, the smaller the diameter of the filled LCR-EMS, the greater the compressive strength.

### 3.3. Characterization of Density and Compressive Strength a of Lightweight Concrete Filled with LCR-EMS of Different Layers

**Figure 6:**
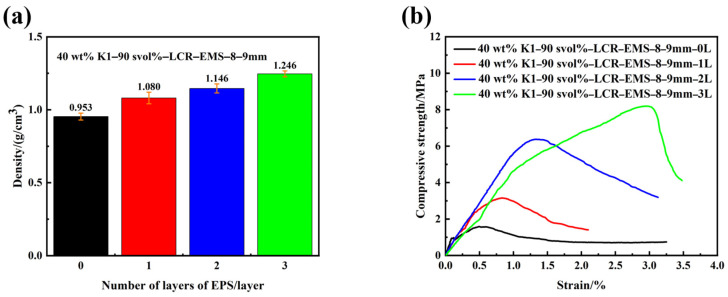
Density (**a**) and compressive strength (**b**) curves of lightweight concrete filled with different layers of LCR-EMS.

The improved performance of single-component LCR-EMS inevitably leads to an increase in the mechanical properties of multi-component lightweight concrete. In order to investigate the effect of reinforcing body layers on the compressive strength of multi-phase composite lightweight concrete, LCR-EMS with an inner diameter of 8–9 mm was used in this series of experiments, and the stacking volume fraction of LCR-EMS added to the matrix was 90%. One, two, and three reinforcing layers were selected and a control group of EPS was established. Figure 6 shows the density and compressive strength curves of lightweight concrete filled with different layers of LCR-EMS. It can be seen from the figure that the variation in the number of LCR-EMS layers also affects the density and compressive strength of concrete. As the number of LCR-EMS layers increases, the density and compressive strength of lightweight concrete increases. When three layers of LCR-EMS were used as filler material, the density and compressive strength of the concrete were 1.246 g/cm^3^ and 8.19 MPa, respectively. The compressive strength of lightweight concrete increased with the increase in the number of layers of LCR-EMS. In particular, compared to the compressive strength of EPS-filled concrete, the compressive strength of filled reinforced layers increased by 98.1% (one layer); 300.6% (two layers); and 415.1 (three layers), respectively. As the number of layers of LCR-EMS increases, the trend of increasing compressive strength improvement of lightweight concrete becomes more and more evident. This is due to the fact that as the number of layers increases, the lightweight calcium carbonate/epoxy structure becomes more robust and less susceptible to damage under stress. The increase in the compressive strength of the filler also benefits the compressive properties of the overall composite.

### 3.4. Characterization of Density and Compressive Strength of Lightweight Concrete Filled with Different HGMS Volume Ratios in the Matrix

**Figure 7:**
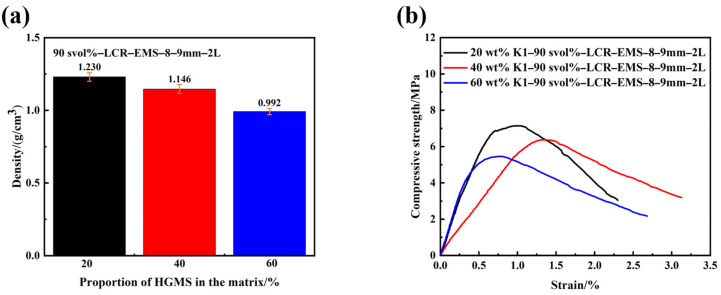
Density (**a**) and compressive strength (**b**) curves of lightweight concrete filled with matrix of different HGMS volume ratios.

In addition to the stacking volume fraction, number of layers, and internal diameter of LCR-EMS filled in concrete affecting the performance of lightweight concrete, the matrix composition also affects the performance of lightweight concrete. In this series of experiments, K1-grade HGMS and cement mixed with volume proportions of 20%, 40%, and 60% were selected as the matrix for lightweight concrete. Figure 7 shows the density and compressive strength curves of lightweight concrete filled with a matrix of different HGMS volume ratios. From the figure, it can be seen that the filler content of HGMS in the matrix also affects the density and compressive strength of lightweight concrete. Due to the lightweight nature of HGMS, the overall density and compressive strength of the matrix decreases as the filler content increases. The density and maximum compressive strength of the concrete were 0.992 g/cm^3^ and 6.01 MPa, respectively, when the HGMS filling content in the matrix was 60%. The compressive strength of lightweight concrete decreases with increasing HGMS content in the matrix. The decrease in the compressive strength of lightweight concrete with the increase in the proportion of HGMS in the matrix is mainly due to the fact that HGMS is a tiny, hollow, spherical powder, which is inherently self-lubricating and non-absorbent. Due to the smooth surface of HGMS and insufficient bonding with cement paste, it leads to more brittle defects in the concrete.

### 3.5. Cross-Section SEM and EDS Mapping of Lightweight Concrete

**Figure 8:**
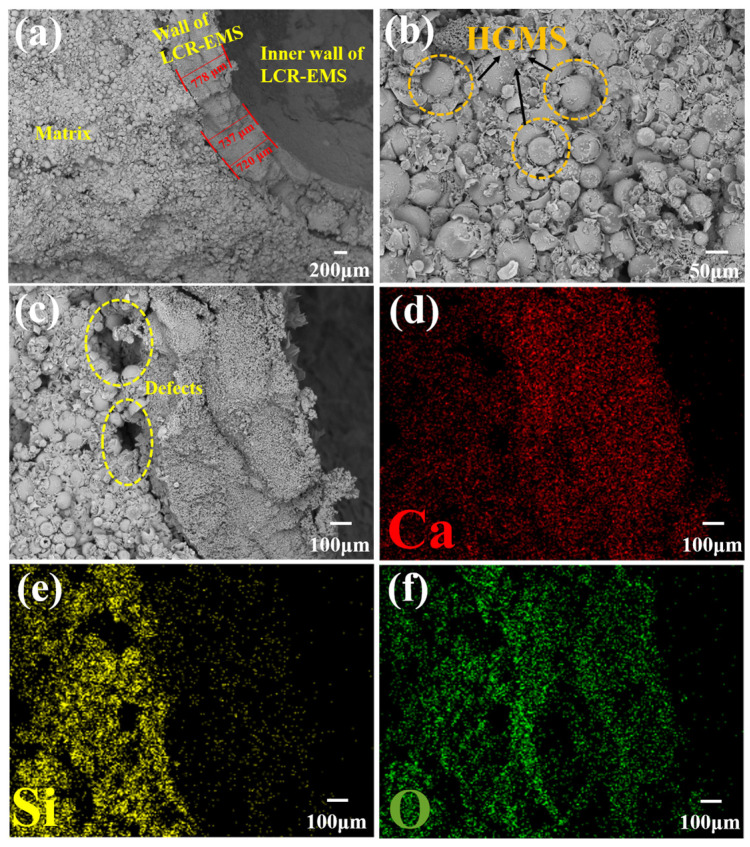
Cross-sectional SEM and EDS mapping of lightweight concrete. (**a**) Lightweight concrete profile at low magnification including matrix, LCR-EMS inner wall and wall thickness. (**b**) Matrix SEM of lightweight concrete. (**c**) High magnification profile of lightweight concrete. (**d**) Mapping of the Ca element. (**e**) Mapping of the Si element. (**f**) Mapping of the O element.

Figure 8 shows the SEM and EDS mapping of the lightweight concrete. From Figure 8a, it can be seen that the average thickness of the LCR-EMS-2 layer can be up to about 745 μm, but there is still a clear demarcation line between the ball and the matrix; but due to the material variability of the ball wall and the matrix and the human factor during the filling process leading to the bonding not being close enough, which leads to the compressive strength of the lightweight concrete, the improvement is not much. Figure 8c also shows that there are still some defects and holes inside the lightweight concrete. Figure 8c–f show the EDS mapping of the lightweight concrete, from which it can be seen that the elements in the mapping are consistent with the elements contained in the raw material.

### 3.6. Compression Resistance Mechanism of AR-EMS Filled Composite Lightweight Concrete

**Figure 9:**
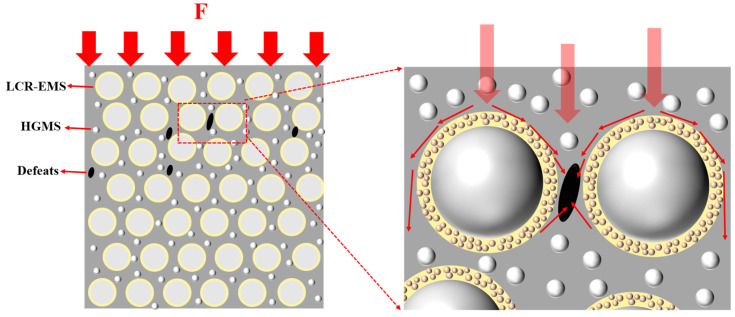
Compressive mechanism of AR-EMS filled lightweight concrete.

Figure 9 shows the compressor mechanism of LCR-EMS filled lightweight concrete. From the figure, it can be seen that when the concrete is compressed by an external force, the force is quickly transferred from the concrete matrix to the LCR-EMS, and then from the LCR-EMS to the matrix or to the next LCR-EMS. The defects of the lightweight concrete are mainly divided into two parts, one is the pore defects in the matrix, and the other is the LCR-EMS. The compressive strength of the whole lightweight concrete depends not only on the strength of the matrix, but also on the compressive strength of LCR-EMS. If the compressive strength of the LCR-EMS itself is very poor, then when the force is transferred to the ball a stress concentration will immediately occur, and thus the damage will start from the LCR-EMS and then be transferred to the matrix, which will eventually lead to the failure of the material. The same phenomenon of material fatigue failure occurs when there are many defects in the matrix. Once the destructive force exceeds the maximum compressive strength of the concrete, the lightweight concrete will fail.

## 4. Conclusions

Calcium carbonate-reinforced LCR-EMS is obtained by adhering calcium carbonate powder to EPS using the “balling method”, and lightweight concrete is obtained by mixing LCR-EMS with cement, hollow glass microspheres, etc., using the “molding method”. Lightweight concrete has different properties depending on the influencing factors. The addition of hollow glass microspheres to the filler can further reduce the occurrence of defects and thus improve the mechanical properties of the concrete. The compressive strength of lightweight concrete was negatively correlated with the stacking volume fraction of LCR-EMS, the initial inner diameter of epoxy composite spheres, and the filling amount of HGMS in the matrix. However, it is positively correlated with the number of reinforcing layers of LCR-EMS. When three layers of LCR-EMS were used as the filler, the density and compressive strength of concrete were 1.246 g/cm^3^ and 8.19 MPa, respectively. The density and maximum compressive strength of the concrete filled with a 90% stacked volume fraction of LCR-EMS were 1.146 g/cm^3^ and 6.37 MPa, respectively. Compared with the lightweight concrete filled with 90% EPS, the density increased by 20%, but the compressive strength increased by 300%. A hard-shell structure can be formed by encapsulating expanded polystyrene foam beads with calcium carbonate powder. The more layers there are, the more stable and solid the hard-shell structure becomes, thus making it easier to resist external destructive forces. The reduction in the diameter of the filled LCR-EMS results in fewer pores and fewer defects between them, which ultimately increases the compressive strength of the concrete.

## Figures and Tables

**Table 1:** Lightweight concrete samples with different parameters.

Sample	HGMS (wt%) in Filler	AR-EMS (svol%)	AR-EMS’s Layer	AR-EMS’s Diameter
1	K1–40	0	2	8–9
2	K1–40	20	2	8–9
3	K1–40	40	2	8–9
4	K1–40	60	2	8–9
5	K1–40	80	2	8–9
6	K1–40	90	2	8–9
7	K1–40	90	2	10–11
8	K1–40	90	1	8–9
9	K1–40	90	3	8–9
10	K1–20	90	2	8–9
11	K1–60	90	2	8–9

## Data Availability

The data presented in this study are available on request from the corresponding author.
